# Narrative Economics, Public Policy and Mental Health

**DOI:** 10.1007/s11482-022-10109-0

**Published:** 2022-10-29

**Authors:** Annie Tubadji, Frédéric Boy, Don J. Webber

**Affiliations:** 1 Economic Department, Swansea University, Swansea, UK; 2 University College London, London, UK; 3 Sheffield University, Sheffield, UK

**Keywords:** Culture based development, Cultural narrative, Narrative economics, COVID-19, Public policy, Health, Cultural hysteresis, Shocks

## Abstract

General public’s mental health can be affected by the public policy response to a pandemic threat. Britain, Italy and Sweden have had very distinct approaches to the COVID-19 pandemic: early lock-down, delayed lock-down and no-lock-down. We develop a novel narrative economics of language Culture-Based Development approach, and using Google trend data for seed keywords, death and suicide, we reach two main conclusions: (i) while countries had a pre-existing culturally relative disposition towards death-related anxiety, the sensitivity to the public policy towards COVID-19 was also country specific; (ii) however, significant spillovers from one specific national lockdown public policy to another country’s mental health are identified.

Under the influence of one and the same negative shock, the Covid-19 pandemic, three different European countries adopted a very different public policy response. Generating in essence a natural quasi-experiment with regard to public policy response to pandemics. In Italy and the UK, public policy switched (with different speed and timing in the response) from “business as usual” to “complete lockdown”. In Sweden, they endured the “business-as-usual” policy. Was this a purely economically relevant decision and was it just a matter of internal policy or did the behaviour of every country affected anyhow the rest of the world?

Firstly, within each country, the effect is related to was is called local resilience. The recent years marked with series of economic crisis had left us with a new term of importance in economics – economic reselienbce, which in essence is the durability of an economy to negative shocks (Bristow & Healy, 2020; Reggiani et al., 2002). We argue however that in fact another aggregate resilience – the psychological one – is of a much greater importance for handling any – economic or health – crisis. That is why maintaining the faith of people in the public policy is an instinctively felt necessity (Norpoth et al., 1991;  Goldberg & Richey, 2020; Ponticelli & Voth, 2020). We venture into documenting why this is so.

Mental health exists on a spectrum of normality (Spijker et al., 2010; Gotts et al., 2012). The ability to maintain an individual’s (or a societal’s aggregate) mental health around that spectrum’s central value is defined as psychological resilience (Fletcher & Sarkar, 2013).

The public policy towards managing the scarce economic resources and our public goods such as our nations’ health, are causing what an economist would call an economic enodeneity of psychological resilience. Put differently, psychological resilience is partially dependent on the economic and public policy implemented in a country (Cornum et al., 2011).

The reason for this is our deeply rooted survival drive which is the main evolutionary concern and fear for survival is one of the most important elements of the human psychological state (LeDoux, 1995; Akerlof & Shiller, 2010; Kahneman & Tversky, 1979). Therefore, the central anxiety due to fear for survival is naturally the psychological response to the current COVID-19 pandemic.

This means that the social context created by the public policy is a source either of increase in public anxiety or of better psychological resilience to survival threats. Clearly, socioeconomic context is a function of the manner in which life is organized and the security network to which people rely on for their survival (Castells, 1996; Kloosterman et al., 1999; Tubadji et al., 2020a). This importance of public policy however has not been recognized with regard to the general public’s mental health.

The prominent work of Michel Foucault (1954, 1961) has opened an important debate about the importance of public mental health policy in terms of how mental institutions are handled. This approach has been widely embraced (Frank and McGuire, 2000). However, the importance of general public policy for general public health is not recognized as a clear question worth monitoring and planning.

We elaborate a filigree quantitative analytical approach relying on the Culture Based Development (CBD) paradigm (Tubadji, 2012, 2013) which allows us to study through the emerging narrative economics of language method (Tubadji, 2020a; Tubadji & Pattitoni, 2020) how public policy affects public mental health.

The novelty of this paper is that it applies this method to analyze the impact of public policy on mental health. The CBD paradigm seeks to explain economic choices that are subject to cultural bias. In this case, the choice under analysis is the public policy decision between “business as usual” and “complete lockdown”. The narrative economics of language quantitative method (Tubadji, 2020b) is inspired by the father of behavioural economics Herbert Simon’s treatment of Zipf’s law of the distribution of words (Simon, 1955) and the recent epidemiology of language approach in narrative economics (Shiller, 2017, 2019). CBD combines these two approaches in order to study the cliometrics of language^1^ as a source of statistical record for the thinking, meaning making and history of ideas. In terms of the intensity in the social discourse, the CBD approach postulates that the thinking, meaning, making and history of ideas exists in a positive relationship to Zipf Law, which drives the statistical relationship of language with economic events and processes over time (Tubadji, 2020a). We contrast the search frequencies of two keywords, “death” (as a proxy for anxiety from death) and “suicide” (as a proxy for the propensity to seek death), on the Google search engine both before and during the COVID-19 pandemic. We analyzed the public policy of lockdown as a treatment that induces changes over time in the frequency of search for these words, which reflects the intensity of mental distress experienced in society under the fear of mortality from the COVID-19 virus. Our findings point towards public policymaking as a powerful endogenous source of information that affects public mental health.

The structure of this paper is as follows. “Economic Resilience and Socioeconomic Context” discusses the definition of psychological resilience and briefly reviews the literature on mental health, public policy, discourse and meaning. “Culture Based Development: Psychological Resilience and Public Policy” summarises the regional economic literature on economic resilience of places. “Data” offers the CBD approach to identifying the causal link between public mental health resilience and public policy. “Method” describes our data and method, which relies on the use of linguistic statistics from Google trends and econometric methods for causal inference. This section offers our results and findings. “Results” concludes and draws policy implications.

## Psychological Resilience, Mental Health and Public Policy

Psychological resilience has been defined in psychology at the individual level, primarily because of its proximal implications and impacts on daily life (Fletcher & Sarkar, 2013; Haddadi & Besharat, 2010). Every individual is entitled to the right to maintain their psychological resilience, and this is a basic need and human right guarantee offered by the UN and most national constitutions (Malkina-Pykh & Pykh, 2013). However, in policy evaluation, the individual is no longer the focus of attention. Mental health is considered differently and, typically, it is discussed with regard to the point of provision for institutionalized mental health care and is discussed in this manner even in some of the most social-behaviour minded studies (Dear et al., 1979; Frank & McGuire, 2000; Hatzenbuehler, 2010; Knapp et al., 2011; Wahl, 2003).

The relationship between public policy and a population’s mental health through the construction of socioeconomic context and discourse has been raised in the prominent work of Michel Foucault (Foucault, 1954, 1961). Foucault belongs to a constructionist philosophical school that assumes that all ideas and meaning are recorded in the language of the discourse (Derrida, 1967; Derrida & Dutoit, 1992; Tubadji, 2020b). Foucault’s example of this linguistic recording is based specifically on the language about madness and mental health. He explains how the mentally ill have been treated differently through public policy in different historical periods, and how this dramatically and negatively has affected their quality of life, especially institutionalized rather than allowed to remain in a social setting. Foucault’s reasoning has been particularly influential on the anti-psychiatry movement (Crossley, 2006) and this was reflected in the public policy shift towards dehospitalization (Haveman, 1986), which is a policy change that has met with mixed sentiments across space (Stavis, 2000; Sercu & Bracke, 2016). We argue here that Foucault’s deconstructionist theory is not only relevant for his topic of interest, clinical mental health, and that public discourse also affects the mental health of the general public.

Foucault’s considerations on the interplay between mental health and policymaking focused on the sufferings of an isolated vulnerable minority in the general population. The present study proposes a novel view that the Foucaultian constructionist mechanism has general applicability for the association between mental health and policymaking of the entire population. Our argument stems from the domain of positive psychology where every individual’s mental health status is a finely balanced dynamic system that exists on a spectrum of normality (Seligman, 2002; Yates & Masten, 2004; Brunzell et al., 2016) and the mental health of every member of society is potentially subject to change under conditions of stress (Babazono et al., 2005). This includes those conditions of stress that are induced by the national-level context and socioeconomic narrative, such as expression of social unrest, revolts and voting patterns, which reflect contemporary and evolving socioeconomic narratives (Benabou & Tirole, 2006, 2009, 2011, 2016). It has also been noted that episodes of social unrest are closely related to public policy (Hirschman & Rothschild, 1973). The COVID-19 pandemic, and its concomitant high level of uncertainty, has created the opportunity to explore the relationship between measures of aggregate mental health and socioeconomic public policy.

### Economic Resilience and Socioeconomic Context

Resilience is not consistently defined or consistently measured in economic terms. Outside of the fields of psychology and physics, the term resilience gained recognition first in ecological science where it was adopted to explain the fluctuation of animal populations (MacArthur, 1955). Subsequent ecological studies of resilience grew significantly (Neubert & Caswell, 1997; Reggiani et al., 2002) and one of the modern definitions of resilience states that it refers to “the ability of an entity or system to “recover form and position following a disturbance or disruption of some kind” (Martin, 2012: p. 4). Some of the most prominent approaches to economic resilience can be found in Nijkamp (1999), Reggiani et al. (2002), Bristow and Healy (2020) and Östh et al. (2018), which maintain concerns predominantly with the economic rather than the socioeconomic aspects of resilience. The use of resilience as a concept that connects the economy with aggregate mental health has not been attempted thus far even though the link is evidently clear. We summarise that in socioeconomic terms, Psychological Resilience of a Population (PRP) is a stability of the general public’s mental health state that ensures the productive operation of the economic system.^2^ We argue that public policy can significantly affect the aggregate mental health and its resilience in any locality.

Complexity theory argues that context is exceptionally important for the entire economic system. Context is a major factor underpinning entrepreneurial research, and the cultural historical institutional roots of a local context determines entrepreneurial success in a path-dependent manner (Acemoglu & Robinson, 2010, 2012). Diversity and entrepreneurship studies show that contemporary contexts affect the efficiency of a team and its ability to be innovative and achieve success (Brunow & Nijkamp, 2018; Brunow et al., 2015). The more general cultural context is a source of multiple complex interactions in the socioeconomic system that vary across geographies and over time. These interactions made Nijkamp (2007) reason that no economic system can be studied successfully if the researcher assumes the *ceteris paribus* condition with regard to the cultural context.

Context and psychological characteristics (particularly personality traits) have been studied thoroughly in the literature as two closely interlinked entities. Economists have embraced the quantification of the Big Five personality traits for the study of the psychological milieu of places across Europe and the USA (Rentfrow, 2014; Schmitt, 2004; Steel et al., 1997). Modern entrepreneurship studies use the Big Five data as an indirect way to quantify the cultural contexts in which entrepreneurs are embedded (Fritsch et al., 2019, 2020; Obschonka et al., 2013). These studies align with earlier research employing the World Values Survey to explore daring attitudes as proxies for a psychological culture that is conducive to innovation (Shackle, 1949; Tubadji et al., 2020b).

The influence of context on socioeconomic behaviour in relation to anxiety has a deep evolutionary behavioural explanation. Although the importance of socioeconomic context was suggested first in relation to financial behavioural economics (Akerlof & Shiller, 2010), we argue from an evolutionary perspective that people are another type of herd animal. It is possible to draw parallels with populations by studying herd animals who effectively decrease their anxiety from threats by using the herd as a signalling tool to identify the presence of a danger (Hall, 1966). Therefore, for humans it is both rational and instinctive (cognitively biased) to choose to adopt a herd behaviour during an external shock to the system, such as a pandemic (Kahneman, 2011). In line with Prospect Theory, an individual’s alignment with a cultural homogenous herd (‘love for birds of the same feather’, McPherson et al., 2001) during normal times and positive shocks is likely to be observed in much greater intensity when experiencing greater uncertainty due to negative shocks, such as the COVID-19 pandemic. This instinctive evolutionary behaviour is the driving mechanism for psychological resilience ensuring survival when under threat. We argue here that humans not only learn about danger from the signalling behaviour of the herd, they can also use rational public policy to decrease anxiety-causing threats through their highly effective socialized communication skills. Therefore their anxiety levels respond to public policy.

The present study explores how such evolutionary psychological resilience mechanism generates a response to various culturally relative public policies across different countries during the Covid-19 pandemic. We advocate the use of a specific cultural economics paradigm that is consistent with a novel linguistic empirical approach for the analysis of the impact of public policy, as described in the next section.

## Culture Based Development: Psychological Resilience and Public Policy

The Psychological Resilience of a Population (PRP) should be regarded as a spatially evolving endogenous element that affects our response to and was effected by the COVID-19 pandemic. This study examines the mental health effects of the Covid-19 pandemic directly, yet these mental health effects represent a secondary source of influence in the public’s reaction to all further public policy interventions.

The main operational definitions of the CBD paradigm (Tubadji, 2012, 2013, 2020a) relevant to this study are culture, cultural capital, cultural milieu and cultural gravity. These operational definitions have been defined and analyzed elsewhere, but can be summarised as:

1. Culture is the set of beliefs and attitudes that exist in a place and inspire choice and action (Tubadji, 2013).
2. Cultural capital is the endowment of material and immaterial assets that quantify the stock of culture available in a place. Cultural capital has two main dimensions: cultural heritage (assets constructed in and inherited from historic periods that create path dependence) and living culture (assets that are being constructed in the present and represent cultural innovations and cultural change in the place) (Tubadji et al., 2020a; Tubadji & Montalto, 2021).
3. Cultural milieu is the amalgam of predominant attitudes that form a context (or general discourse) which is created by the culture of a place and in which the rest of the socioeconomic processes are embedded (Tubadji et al., 2020a).
4. Cultural gravity is the appeal of a place that attracts and concentrates human capital, and is based on the type of cultural milieu that the place has (Tubadji & Nijkamp, 2015).

From this CBD paradigm point of view, mental health is part of the cultural milieu; it creates the current context and records in the cultural heritage of a place. Therefore, preservation of a locality’s healthy, favourable and attractive cultural milieu maintains care not only for the current members of the place but also for its regional development.

From a methodological perspective, the Narrative Economics of Language (Tubadji, 2020a) is a novel CBD analytical method that is used to investigate the link between socioeconomic development and the context of thinking, meaning and ideas through the information recorded in the statistical characteristics of language. This CBD method is based on two priors: (i) statistical reasons to consider language as a reliable evolutionary record of thinking and meaning and (ii) empirical advantages for analyzing relationships between phenomena using time series of detailed big data. Thus, this approach is motivated by two considerations:

1. Zipf’s distribution^3^ (Zipf, 1935, 1949) was noted by Herbert Simon to underlie the distribution of many socioeconomic phenomena, such as the growth of cities, population and firms. The CBD narrative economics of language perspective builds on Simon’s (1955) observation and suggests that the statistical dominance of a word among a distribution of words, which accords to the Zipf Law ranking, also corresponds to the dominance of the narrative in the contemporary public discourse. Similarly, psychology uses words as verifiable signifiers of emotional states (such as anxiety for mental health) (Löwe et al., 2008).^4^ Thus, the CBD method considers words that are signifiers for a certain emotional state and that have a higher Zipf ranking to have stronger prevalence of the respective psychological trait in the local cultural discourse (or context).
2. Publicly available big data from online sources, such as Google, Instagram, Facebook and Twitter, provide records of information in granular time-series form (see for instance Banerjee, 2018). For instance, Google search volume data can be mined for any keyword and timestamped for every 7.95-min period over the past 15 years. We use this data here. Long time series of high frequency sample data provide an opportunity for causal econometric inference and predictions. It can be used for historical and continuous monitoring and for the forecasting of the public response to endogenous and exogenous treatments, such as a pandemic crisis. Above all, this is real behavioural data, which outsmarts any simulation model and allows for real-time analyses of public opinion and reactions to any public communication and economic policy.

The CBD paradigm and its novel narrative economics of language quantitative methodology is applied below in the case of the use and communication of public policy and its impact on mental health in the times of the COVID-19 pandemic. Our data, analyses and interpretations reveal implications for policymakers and underscore the value added that the CBD linguistic narrative economic methodology offers in terms of gaining fast and reliable insights at a low cost, which were previously not easily available to the researcher or the policymaker.

To position the value added of the above outlined CBD take in the context of the literature, we should consider that the impact from the shock of COVID-19 was examined mostly from the point of vie wof the effect of the shock on within country level. Namely, the effect of the shock was studied among others by Morrison et al. (2022) for the case of New Zealand, by Greyling et al. (2021) for the case of South Africa, or comparing the effect of the shock in Europe versus the United States (see for instance Brodeur et al., 2021). Instead of focusing on the effect from the shock, the CBD approach focuses on the analysis of the impact from different public policy treatments under the effect of the same shock (COVID-19). Thus, the CBD approach adds a unique empirically-operationalized comparative institutional angle to the analysis of the link between public intervention in crisis and the well-being/mental health of the population. The CBD approach allows to empirically focus the attention on the cultural institutional heterogeneity in the public policy response to the COVID shock across otherwise mostly homogenous (or largely cohesive) economically Europe.

## Data

Our linguistic dataset contains the frequency of keyword searches using Google trends. The keyword of primary interest is the word ‘death’, which we claim to be the best proxy for the mental state of anxiety from death during the Covid-19 pandemic. We also obtained the frequency of the word ‘suicide’ as a control for the opposite mental state of anxiety motivated by the desire for death. Finally, we obtained frequencies for a word that is neutral to anxiety, ‘chair’, in order to control for increases in keyword search behaviours due to the increased need to stay at home during the lockdown, and this enables us to distinguish between the increase in the use of the internet during the lockdown and the increase in the levels of anxiety as an experienced mental state.

Previous research shows that Google keyword searches vary depending on the day of the week (Boy & Tubadji, 2020) and therefore it is present also in our Google trend data. We employed day of week dummy variables in our regressions, which we use in the sense of fixed effects to capture the weekly seasonality in the data, but also use these dummy variables to reconfirm and analyze differences in experienced anxiety during the week.

It is noteworthy here to clarify the differences and similarities that previous work with big data from Google and similar sources has done with regard to COVID-19. The literature with big data divides importantly in two types: using the internet search frequency for cultural linguistic markers to approximate the mental state in the big data (see for example Brodeur et al., 2021) or analytical approach to identifying the mental state relying on sentiment analysis og internet posts. The first approach has the advantage that it is the directly observed intensity of the mental state marker, while the sentiment analysis is the inferred by the researcher second-guessed mental state of the observed electronically interacting population (which may cary a lot of error in the identification of the mental state). Meanwhile, the use of a single proxy variable (such as a key word frequency) is always potentially narrowing down the information, while relying on a pool of indicators (as the sentiment analysis does) may have the advangae of more fully quantifying the mental state. Our study adopts to the first, more clearly identified measurement approach. This approach has been validated also through the comparison between the internet big data search frequencies and the self-reported survey on mental health for the UK (see Boy et al., 2022).^5^

We augmented the linguistic big data with official statistics that record the number of deaths due to COVID-19 within each country of interest. Next, we added information about the dates for the imposition of the lockdown rule, which varies across countries. These dates were 12/03/2020 for Italy and 23/03/2020 for the UK. We created a dummy variable for each day, where the pre-lockdown periods were assigned a value of zero and a value of one for the lockdown period. We interacted these dummy variables with a time trend in order to obtain the policy impact interaction terms.

We considered the day of the WHO statement on the meeting of the International Health Regulations related to the COVID-19 pandemics, which was released on 23^rd^ January 2020. This date has a particularly strong association with the Swedish data, which experienced a spike in keyword search behaviour for the word ‘death’ on this particular day. Finally, we constructed a model where the variation in the number of keyword searches in the linguistic data was explained by mortality data and the public policy decision to undertake a lockdown.

## Method

Culture is considered as a “programming of the mind” (Hofstede & Hofstede, 1991; Signorini et al., 2009) and is our motivation to claim that cultural differences in the lockdown policy will affect the general public’s mental health. The rationale to believe that public policy has an effect on mental health is rooted in the evolutionary behavioural economics perspective that public behaviour is a response to the herd signalling uncertainty and anxiety (Akerlof & Shiller, 2010; Hall, 1966). In the contemporary era of a free flow of information, the public can observe, compare and contrast what their own and other nations do as a response to the pandemic. Our herd is ultimately beyond our cultural national herd and instinctively we perceive ourselves as part of the universal human herd. Thus, from a Culture Based Development viewpoint, we test three hypotheses relating to society’s mental health behavioural response to the COVID-19 pandemic:

- *H01:Public policy on lockdown decreases public anxiety from death in the country of residence.*
- *H02:Increases in the number of reported deaths escalates public anxiety from death in the country of residence.*
- *H03:A country’s decision to employ a lockdown policy increases public anxiety from death in other countries that have different lockdown policy regimes.*

The above hypotheses rest on the assumption that public mental health is a function of quantifiable objective factors (such as death rates) and the public’s emotional sensitivity to policy employed for handling the objective factors. This public policy reflects the culture of management of the Covid19 problem within a country, and in relative terms can be expressed as the difference in the time of imposition of a lockdown rule (or its complete avoidance) relative to other countries. The operational model through which we test our working hypotheses is:

1 $$ANXIETY\_FROM\_DEATH=\alpha +{\beta }_{1}LOCK\_DOWN+{\beta }_{2}DEATH\_NUM+{\beta }_{3}X+{e}_{1}$$

where *ANXIETY_FROM_DEATH* is the frequency of search for the keyword ‘death’ on a particular day in a particular country, which our CBD methodology uses as a linguistic signifier for mental health anxiety at the national level. *LOCK_DOWN* captures the nation’s psychological sensitivity to the imposition of a lockdown policy; it is a vector that includes a time trend on a daily basis and a dummy variable equal to one on and after the day of imposing the lockdown, as well as the interaction of these two variables. *DEATH_NUM* is the official number of reported deaths, and this is the salient number that affects the cognition of the public. *X* is a vector of other confounding factors, such as the date of lockdown in neighbouring countries. The inclusion of data capturing the lockdown date of neighbouring countries can be justified from the perspective that it signals the behavioural inconsistency in handling the same anxiety employed by another group of people and their different survival strategies under conditions of uncertainty. Such differences in policy choice and asymmetries in hesitation can raise anxiety further.

First, we employ a difference-in-differences approach using an OLS with time trend where the day of lockdown in the home country is considered a treatment and the effect of the difference-in-differences is the interaction between the daily time trend and the dummy equal to one from the day of treatment onwards (Conley & Taber, 2011). We correct for weekly seasonality and explore the impacts of the national and international lockdowns on anxiety levels as expressed in the keywords. Second, we employed an interrupted time series analysis (ITSA), where the interruption is the national or international lockdown, and used the above-mentioned seasonalities, the lockdown date in home country, and additional lockdowns as confounding factors.^6^

Sweden did not impose a lockdown, and it serves as a control group for the countries who did impose one, such as the UK and Italy. In order to use this natural quasi-experimental setting to explore the public mental health response to public policy, we calculated the daily differences in keyword searches between Sweden, the UK and Italy. This enabled us to explore how these differences in keyword searches changed as a function of the differences in the objective number of deaths between Sweden and the countries that did impose a lockdown and the differences in timing of the culturally-informed imposed lockdown.

To check the robustness of our cultural and linguistic methodology, we applied model (1) to explain the search for the words ‘suicide’ and ‘chair’. The first one is related to our ‘death’ results, but instead of being concerned that death may come early, ‘suicide’ indicates an anxiety associated with the desire for death. Therefore, based on the CBD linguistic method we expect to detect a fall in the search of the word ‘suicide’ while searches for the word ‘death’ would increase, as these two notions signify linguistically the meaning of two opposing mental states. Next, the word ‘chair’ is expected to show, what we have coined as: the ‘IKEA effect’, approximating the “business as usual’ attitude. The word ‘chair’ is a word that is neutral to the daily routine and indifferent to our mental states of interest, and a ‘chair’ is a piece of furniture with a particularly level of high importance for the liveability of a space (Kirkham, 2006).

## Results

**Fig. 1:**
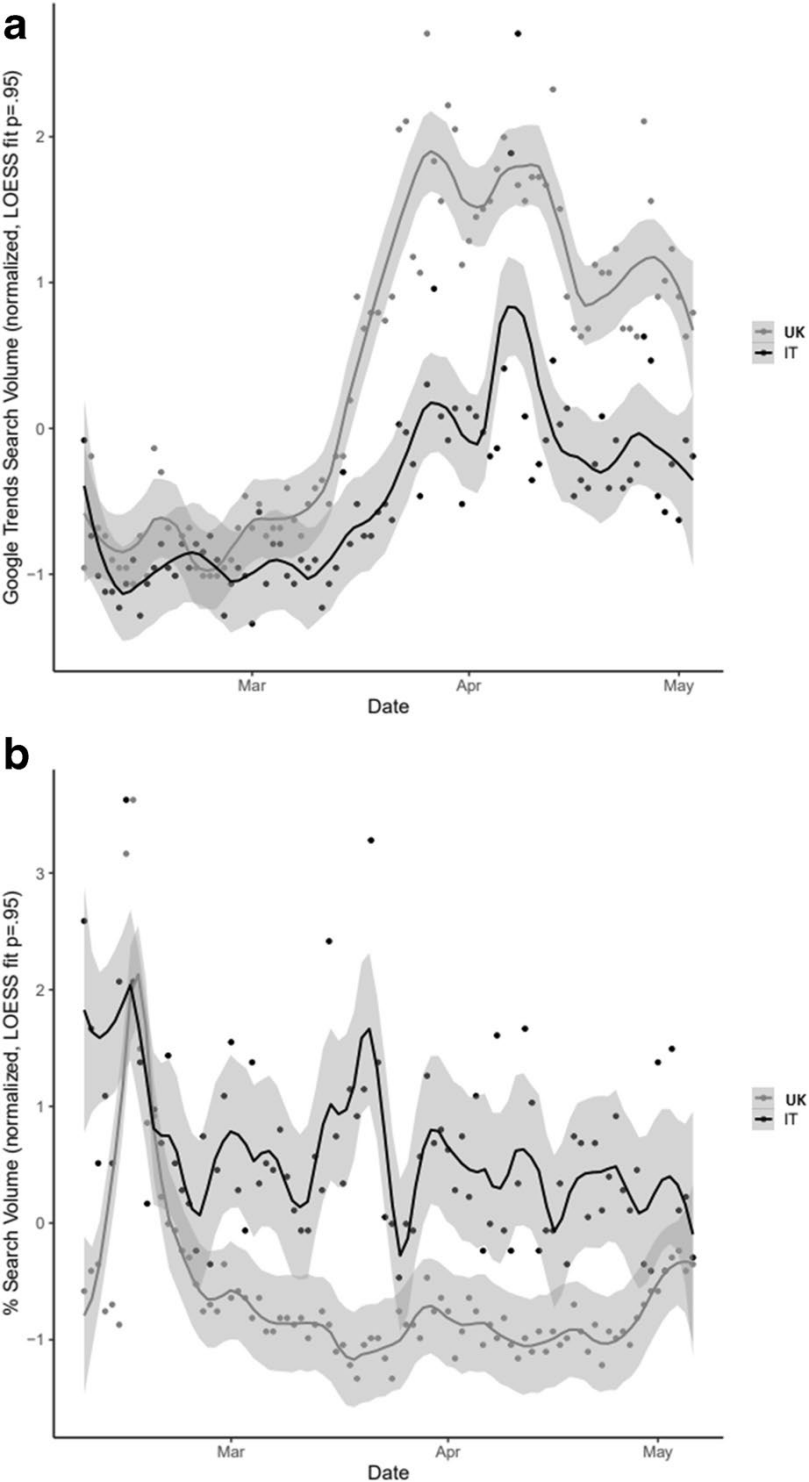
(**a**) Searches for ‘death’ before and during the pandemic (**b**) Searches for ‘suicide’ before and during the pandemic. Note: Figure 1a & b are derived using Google trend data

An initial look at the temporal evolution of the frequency of search for the word ‘death’ demonstrates that this word has grown in interest since the start of the Covid-19 pandemic, as shown in Fig. 1a. This pattern is sinuous and varies in magnitude but the trajectory is upward for both Italy and the UK, which means that the pandemic has generally increased the experienced anxiety on average for both countries. We interpret the differences in the trends observed in Fig. 1a as a reflection of the cultural differences across space in terms of cultural relativity. We also see different magnitudes in the responses to the same treatment across space, and this effect is termed by CBD as cultural hysteresis (Tubadji et al., 2016).

To crosscheck that we are observing a death anxiety effect and not simply a general increase in the volume of online searches due the requirement to stay at home during the lockdown, we compared the trend in searching for the word ‘death’ with the trend in searching for the word ‘suicide’, as shown Fig. 1b. Instead of an upward trend, the search trend for the word ‘suicide’ was downward. This is a confirmation that our linguistic analysis approach captures the public mental state in a constructivist and deconstructionist manner, where notions and mental states build up attitudes towards the reality in a culturally contextualized manner and keywords signify and statistically record the evolutionary development of the context.

A closer look at the tendencies in the three countries under analysis can be gained by comparing descriptive statistics of the variables of interest before and during the pandemic, as shown in the Appendix. We compared keyword searches in the UK and Italy against the control country of Sweden and three main insights emerge. First, the population of Sweden (Italy) searched for the word ‘death’ least (most) often both before and during the pandemic.^7^ Second, the pandemic increased the frequency of search for the word ‘death’ across all countries. Third, the increase in the search for the word ‘death’ was greatest in the UK where it doubled, while in Italy there was only a 50 percent increase in the number of searches for the word” death’. These statistical observations are consistent with CBD-related expectations that there are different response magnitudes to identical negative shocks across space due to an underlying cultural bias (Tubadji et al., 2016). This cultural bias effect needs to be disentangled empirically from the timing of the lockdown effect and from the part that is related to the increase in deaths across the countries under analysis.

### Difference-in-Differences Analysis

A difference-in-differences approach is selected to obtain deeper empirical insight into the preciding descriptive statistics. Table 1 presents the results for within country differences in anxiety from death before and after the implementation of the lockdown policy. Table 1 exhibits three specifications. Specification (1) tests H01 and explores the effects of lockdowns on national levels of public anxiety from death. Specification (2) tests H02 and explores the effects on national levels of public anxiety from death associated with the publically announced number of deaths, which signals uncertainty for life. Comparison of the results in Specifications (1) and (2) helps to distinguish whether the effects hypothesized in H01 or H02 dominates in model (1). Specification (3) augments the model by adding the treatment effect and its temporal and spillover interaction terms, thereby considering the effect of the Italian lockdown on anxiety in the UK and vice versa. This captures international spillover effects and tests H03.

**Table 1:** Death anxiety: public policy, death numbers and spillovers

	Public Policy Effects						Salient Death Number Effects						Spill-Over Effects b/n countries					
	UK			IT			UK			IT			UK			IT		
dep. var	*DEATH ANXIETY*																	
	coef	t-value		coef	t-value		coef	t-value		coef	t-value		coef	t-value		coef	t-value	
*day_id*	0.302	5.90	***	-0.115	-2.90	**	0.296	6.17	***	-0.135	-3.25	***	0.117	5.58	***	-0.125	-2.90	**
*treatment_lockdown_UK*	104.324	2.15	*				229.767	1.89					268.977	2.46	*	-66.164	-0.71	
interaction_lockdown_UK*day_id	-0.867	-1.62					-2.376	-1.62					-3.272	-2.50	**	107.506	0.97	
*deaths_mean_UK*							0.029	1.05					-0.016	-0.62				
*treatment_lockdown_Italy*				-82.274	-3.29	***				-24.892	-0.95		-243.200	-7.20	***	-1.223	-0.91	
interaction_lockdown_Italy*day_id				1.248	4.07	***				0.351	0.97		3.473	7.60	***	0.938	0.75	
*deaths_mean_Italy*										0.030	2.96	**				0.017	1.12	
FE week	YES			YES			YES			YES			YES			YES		
_cons	25.016	9.65	***	56.196	17.25	***	25.123	9.76	***	57.037	17.16	***	29.828	19.38	***	56.793	17.19	***
Num of observations	94			94			94			94			94			94		
R-squared	0.81			0.46			0.81			0.48			0.93			0.49		

The table presents regression for the frequency of search of the word ‘death’. The table presents OLS with fixed effects for weekly seasonality, day detrending, and interaction effects between the day detrend and the lockdown date in the respective country as a direct treatment effect. Spillovers are considered through the additional inclusion of a treatment effect for the other country and the interaction between this additional treatment effect and the day trend

Table 1 offers several insights. First, the early imposition of a lockdown in Italy had a detrimental effect on the mental health of both the Italian and UK populations. Once the lockdown was subsequently imposed in the UK, this decreased anxiety especially in the UK. This result is consistent with CBD assumptions about herd signalling and anxiety effects, as described in Hall (1966): the UK was experiencing increased anxiety because it was observing Italy taking much more intensive precautions under the same pandemic threat. The inconsistency of the UK policy was effecting negatively Italy as well. Across all countries, greater mortality had a positive statistically significant effect on anxiety by increasing both the explanatory power of the model and influencing the rest of the effects in the estimations, and this mortality effect on anxiety was more intense in Italy.

Between country differences in death reporting practices existed (e.g. whether deaths occurred in hospitals, care homes or domiciles), yet what plays a comparable role is the fact that the reported number of deaths is the salient effect of the pandemic that people observe. Thus, the response to the differences in the methods of counting and public announcement of numbers has an important behavioural effect rooted in cognitive biases (Kahneman & Tversky, 1979). The effect of the UK’s decision to introduce a lockdown seems to have decreased anxiety in Italy; our interpretation of this is rooted in a behavioural explanation of signalling confirmation of the chosen strategy for survival.

**Fig. 2:**
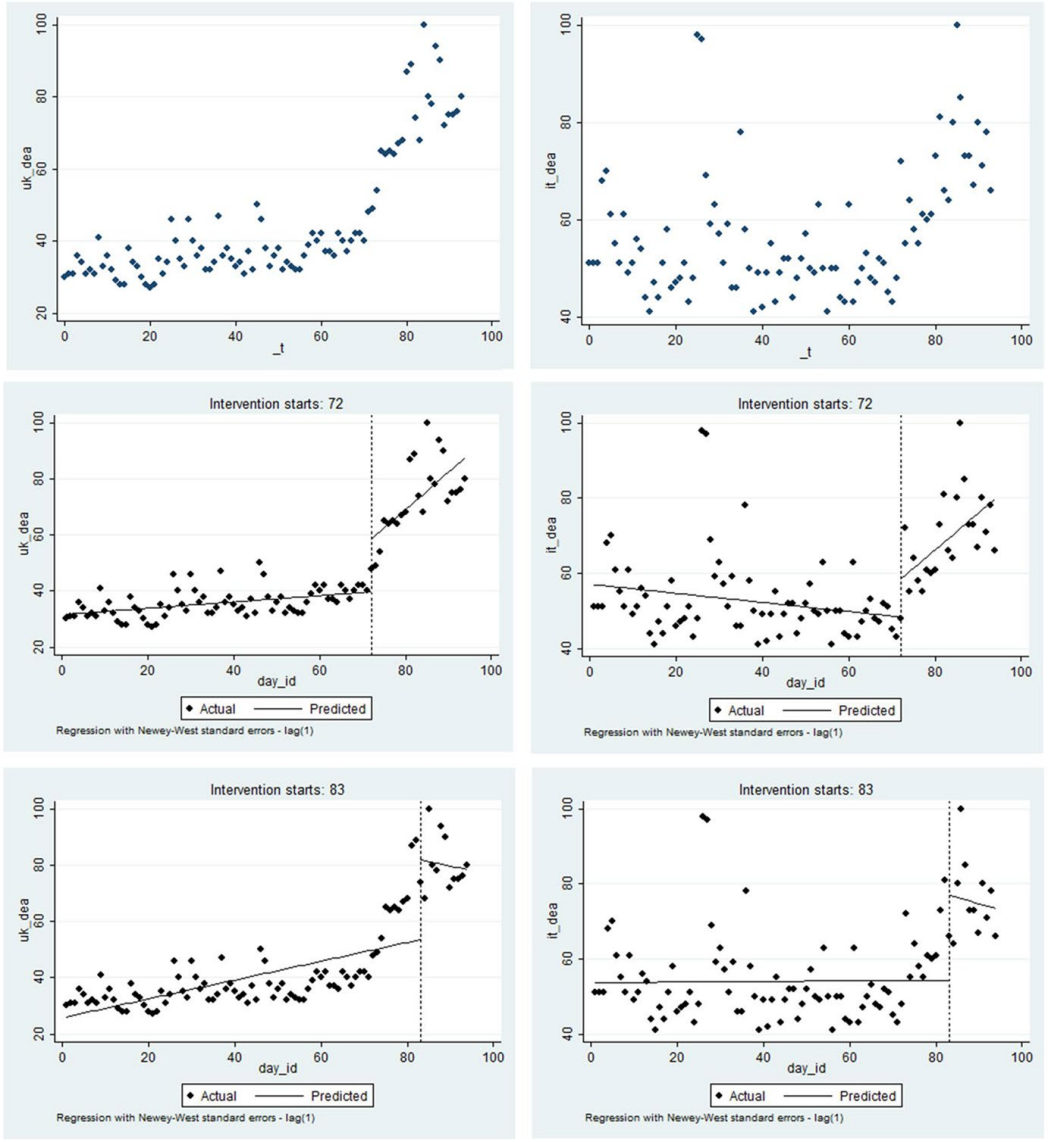
Death anxiety with different lockdown effects: UK vs. Italy. Notes: the left column are figures for the UK while those for Italy are on the right. The first row of figures shows the frequency of searches for the word ‘death’ in Google. The second row of figures considers the day of lockdown in Italy as a treatment for the interruption of the time searches in both UK and Italy (12 March 2020). The third row presents the interruption of the time series associated with the lockdown in the UK (23 March 2020)

Figure 2 provides a complementary examination of the data. Fitted linear trends for pre- and post-lockdown periods are presented for both the UK and Italy. Both countries seem to have experienced a visible disturbance in their linear trends when the lockdowns were introduced. Consistent with Table 1, the interruption of the trend is associated with an increase in anxiety in both countries after the imposition of the lockdown policy in Italy on day 72. Similarly, a decrease in anxiety from death occurred in both countries in response to the imposition of a lockdown in the UK on day 83; we argue this supports our CBD interpretation that the behaviour of related groups serves as a signal for confirming the appropriateness or enhancing the scepticism towards our own survival strategy during times of uncertainty.

**Fig. 3:**
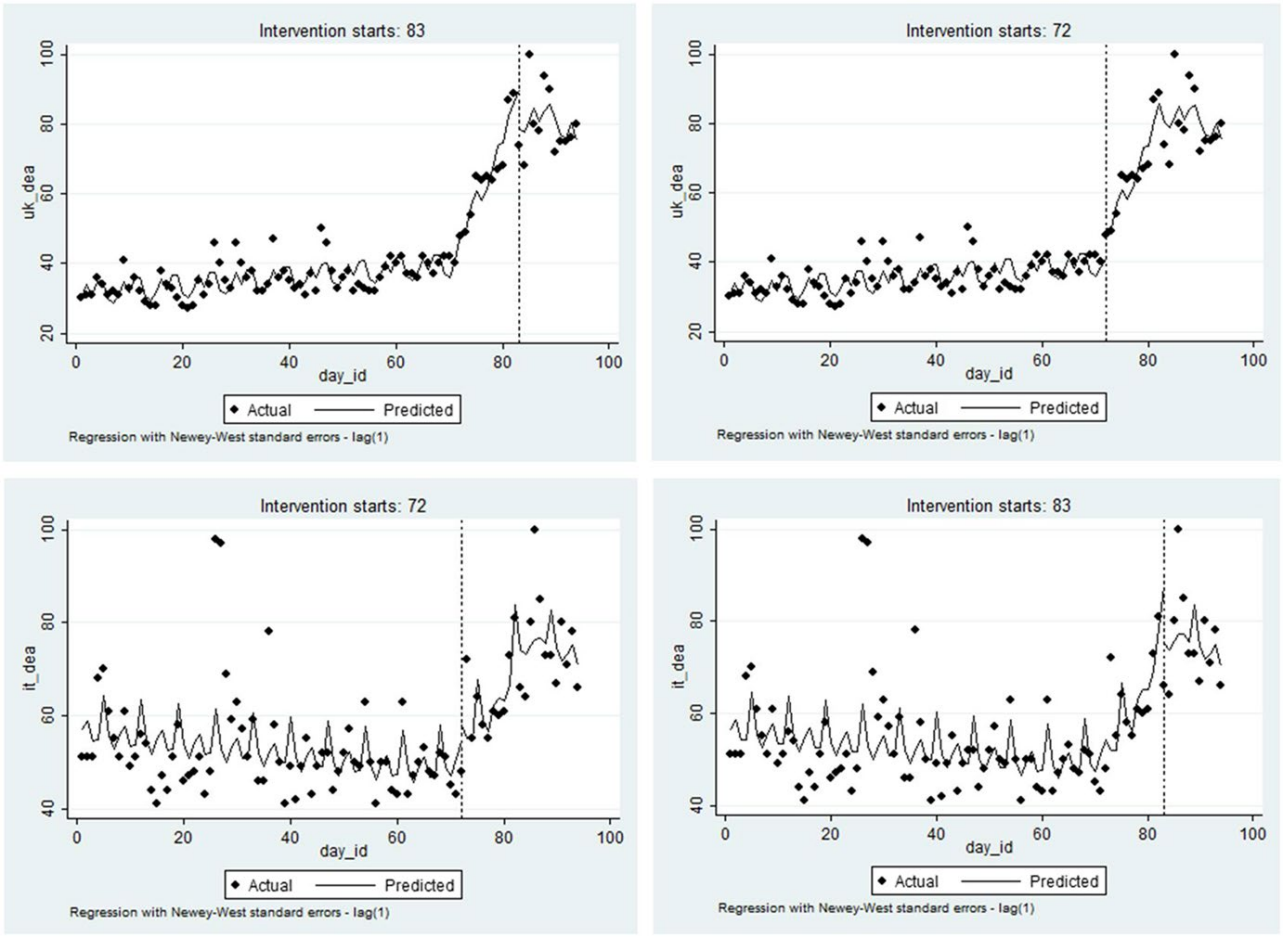
Predicted values of death anxiety as post-estimation from ITSA. Notes: The figure presents the predicted frequency of searching for the word ‘death’ in Google in the UK (first row) and Italy (second row), with respectively national and international (of the other corresponding country) lockdowns

Figure 2 serves to justify the implementation of a policy impact assessment using an interrupted time series analysis (ITSA), which is a special case of the regression discontinuity technique where the treatment is identified at a point of time (Hausman & Rapson, 2018). Our ITSA is implemented with regard to the lockdown treatment in the home country and with regard to the lockdown decision in the other country. In both cases, we considered the alternative treatment date as a confounding factor. Table 2 presents these results^8^ and again reveals the presence of the impact of the lockdown on mental health. The difference in the sign of the effects of the Italian and UK lockdowns is confirmed in this within-method triangulation, as was revealed in Table 1. Plots of the predicted values for both the UK and the Italian cases are supplied in Fig. 3 and reveal trends typical for interrupted time series.

**Table 2:** Interrupted time series analysis for dealth anxiety

	Spec. 1						Spec. 2					
	UK						IT					
dep. var	*DEATH ANXIETY*											
	coef	z-value		coef	z-value		coef	z-value		coef	z-value	
*_day_id*	0.117	4.86	***	0.119	5.08	***	-0.134	-2.63	**	-0.124	-2.44	**
*_day_83*	-5.705	-0.91								5.624	0.67	
_interaction_day_id*day_83	-2.954	-1.74								-1.582	-1.11	
*_day 72*				7.342	3.12	***	5.556	1.18				
_ interaction_day_id*day_72				3.366	6.70	***	0.426	0.33				
*deaths_mean_UK*	-0.022	-0.74		-0.016	-0.65							
*deaths_mean_Italy*							0.016	1.12		0.016	1.21	
*treatment_lockdown_Italy*	-257.606	-8.64	***							-83.450	-1.08	
interaction_treatment_lockdown_Italy*day_id	3.667	9.07	***							1.177	1.14	
*treatment_lockdown_UK*				261.927	2.32	**	65.812	0.56				
interaction_treatment_lockdown_UK*day_id				-3.181	-2.34	**	-0.686	-0.49				
FE week												
2	3.954	1.88		3.728	1.76		1.917	0.60		2.186	0.70	
3	0.673	0.38		0.490	0.28		-2.280	-0.72		-2.090	-0.61	
4	4.608	2.08	*	4.458	2.03	*	-1.903	-0.63		-1.862	-0.61	
5	4.599	2.02	*	4.484	2.03	*	7.898	1.77		8.559	1.93	
6	-0.911	-0.41		-1.141	-0.53		-0.619	-0.13		-0.558	-0.12	
7	-2.001	-0.87		-2.230	-0.98		-3.464	-1.08		-3.440	-1.06	
_cons	30.002	17.91	***	30.112	17.99	***	56.996	15.88	***	56.591	15.87	***
Num of observations	94			94			94			94		
Treated	-2.837	-1.67		3.486	6.95	***	0.292	0.22		-1.706	-1.21	

The table presents an interrupted time series analysis, where the frequency of searches for the word ‘death’ is explained by the treatment, which is alternatively considered to be either day 72 (the 12 March 2020) or day 83 (the 23 March 2020) for Italy and the UK respectively. Spillovers included

It is pertinent to consider these effects when compared to a country that did not introduce a lockdown policy, Sweden serves as this control. The Swedish population has been exposed to mortality information and thus represents an excellent control for isolating and analyzing the effect of the implementation of the public lockdown policy in the anxiety function presented in model (1).

### Sweden as a Control for the Lockdown Public Policy

The estimations using the differences between Sweden and the UK on the one hand, and Sweden and Italy on the other hand, follow the same logic as the estimations in the previous section. We implement three specifications: national effects, mortality effects, and finally cross-country spillover effects. However, in these estimations the dependent variable is the difference between the base country (Sweden) and the treated country (UK or Italy respectively). Results are shown in Table 3 and provide confirmation for the results in Tables 1 and 2 when using Sweden as a control. Moreover, the estimated effects are statistically stronger when attention is focused on the differences across space.

**Table 3:** Sweden as a control group for public policy in the UK and Italy

	Public Policy Effects						Salient Death Number Effects						Spill-Over Effects b/n countries					
dep. var	*DIFF_UK_SE*			*DIFF_IT_SE*			*DIFF_UK_SE*			*DIFF_IT_SE*			*DIFF_UK_SE*			*DIFF_IT_SE*		
	coef	t-value		coef	t-value		coef	t-value		coef	t-value		coef	t-value		coef	t-value	
*day_id*	0.268	3.13	***	-0.144	-1.43		0.249	3.01	***	-0.166	-1.60		0.086	0.91		-0.163	-1.57	
*treatment_lockdown_UK*	34.875	0.61					463.322	2.70	**				364.864	2.68	**	33.752	0.56	
interaction_treatment_lockdown_UK*day_id	-0.033	-0.05					-5.180	-2.50	**				-4.137	-2.54	**	0.187	1.67	
*DIFF_DEATHS_UK_SE*							0.110	2.57	**				0.087	3.34	***			
*treatment_lockdown_Italy*				-95.562	-2.57	**				-39.408	-0.76		0.252	2.60	**	0.030	1.41	
interaction_treatment_lockdown_Italy*day_id				1.435	3.12	***				0.543	0.73					-0.435	-0.51	
*DIFF_DEATHS_IT_SE*										0.033	1.36							
FE week	YES			YES			YES			YES			YES			YES		
_cons	12.099	2.05	*	43.177	6.69	***	12.679	2.17	*	44.167	6.61		16.150	2.91	**	43.949	6.55	***
Num of observations	94			94			94			94			94			94		
R-squared	0.49			0.25			0.51			0.26			0.55			0.26		

The table presents OLS with fixed effects for weekly seasonality, day detrending, and interaction effects between the day trend and the lockdown date in the respective country as a direct treatment effect. Spillovers are considered through additional inclusion of a treatment effect for the other country and interaction between this additional treatment effect and the day trend

Results provide support for H01, H02 and H03. They indicate that mortality as an objective factor dominates the impact on the population’s mental state, which is not void of public policy bias either, as the method of calculation and especially the reporting of numbers are subject to a policy decision making that responds to culture. Although this corroborates the idea that people are generally realistic and rational, it also emphasizes that we suffer cultural biases when acting under uncertainty. People may have been informed about the different ways that deaths were calculated in their country relative to another country, but the numbers reported generated have a similar type of effect. While these results reveal the sensitivity of the population’s mental state to publicly reported deaths, we are not advocating the manipulation of reported figures but are instead underscoring that public policymakers should be aware of the repercussions of its announcements and actions as it plays a significant role in stabilizing a population’s mental health.

### Hourly Effects and Other Robustness Checks

**Fig. 4:**
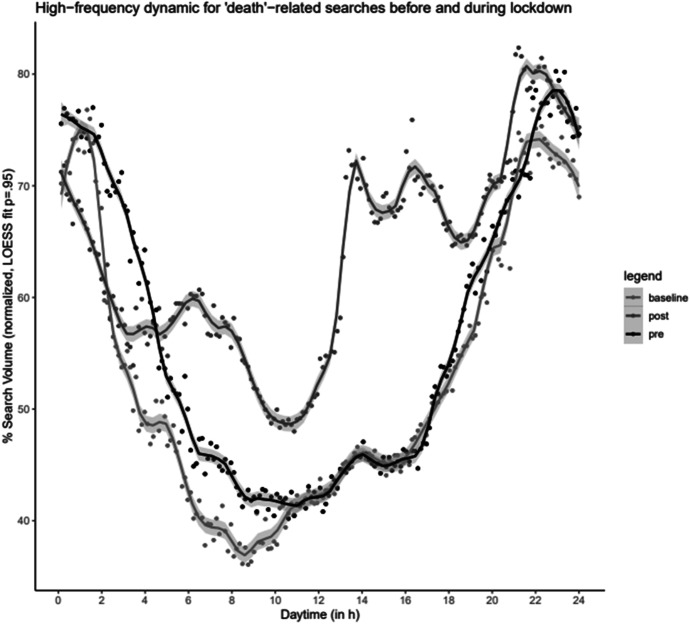
Granular data on daily death anxiety in the UK. Notes: The figure presents the frequency of searching for the word ‘death’ in Google, with baseline representing the pre-pandemic period in the UK, between 00:00 and 24:00 h. Source: Google trend data

To further cross check our findings, we engage in robustness checks of our narrative economics of language approach and its ability to explain the behavioural differences across countries in response to Covid-19 pandemic. A detailed exploration of the hourly profile of searches for the term death during an average day in the UK is presented in Fig. 4. This figure splits the searches into forthnights pre-lockdown and post-lockdown periods and uses the beginning of the pandemic (1^st^-14^th^ January) as a baseline. Figure 4 reveals three noteworthy facets. First, there is a peak in search intensity for the word death between the hours of 20:00 and 02:00.^9^ Second, there is a temporal pattern of keyword search using Google for the word death that existed before and continues after the lockdown. Third, the lockdown policy has smoothed the frequency of searching for the word death in Google during the day, which illustrates more consistent levels of experienced anxiety among the UK population on a daily basis. Nevertheless, the hours leading up to bedtime remained the most difficult for handling anxiety from death.

**Fig. 5:**
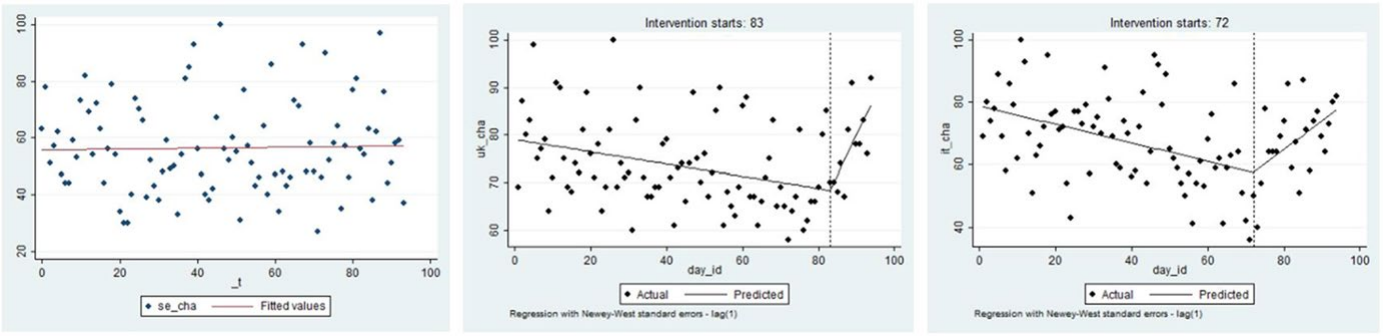
Lockdown policy on business as usual – the IKEA Effect. Notes: The figure presents the daily Google’s relative search frequency for the word ‘death’ in Sweden, the UK and Italy (from left to right) for the period 01/01/2020 to 03/04/2020. The figures for UK and Italy present the day of lockdown and fitted lines for pre- and post-lockdown periods

Figure 5 shows the temporal pattern of the search for the emotionally neutral word ‘chair’ in Sweden, the UK and Italy. For the UK and Italy, we also indicate the time of the lockdown period with a vertical line and fit a line in the observations before and after the lockdown was imposed in the respective country. There is no change in the frequency of search for chairs on Google in Sweden for this entire period. In contrast, the figures for Italy and the UK are less stable and a fitted line suggests a progressive decrease in interest in chairs in the early part of the pandemic before the lockdown and an increase in searches for chair after lockdown. This suggests that people engaged more intensively in online searches after the lockdown.^10^ However, there is no discontinuity in the search trends, as was the case in the frequency of searches for the anxiety-related word” death’. We interpret this as a demonstration of the impact that public policy can have on a population’s anxiety, which is not an identical and spuriously observed tendency across all words. Therefore, statistical explorations confirm this cultural narrative economics empirical approach as a reliable and meaningful tool for analysis. This is an important message in the time of big data, where linguistic evidence is abundantly available but yet to be harnessed for policy impact evaluation purposes.

## Conclusion

Public policy creates a context in which the society operates and is a major factor that contributes to the complexities of socioeconomic decisions and behaviours that are embedded in a place. Context contributes to the explanation of differences in responses and effects of identical conditions across space and to the asymmetric responses to an identical shock. Following this reasoning, this paper suggests a Culture-Based Development approach for the analysis and understanding of the culturally embedded spatially diverse differences in public policy responses towards the Covid-19 pandemic, as revealed in imposition decisions on lockdown policy.

Comparison of the sensitivity i.e. the mental health reaction of the population to the lockdowns in the UK and Italy, and contrasting these reactions with the “business as usual” control case of Sweden, enables the analysis of three main groups of effects. First, we were able to study the effects of the public policy with regard to lockdown in the country where the policy was introduced, as well as its spatial spillovers across border to another country. Both Italy and the UK were intensely present in the international monitoring of the pandemic crisis through their media, and therefore they represent an excellent basis for studying cross-country spillovers. The analysis identified and distinguished the effects from the different public policy implementations and the effects from the objective factor of the number of deaths. The analysis distinguished between the inter-country cultural relativity (the sensitivity of the populations towards the public policies deemed culturally relevant by the institutions in different countries) from the inter-country cultural hysteresis (i.e. differences in the extent of the sensitivity to the same trigger – the COVID-19 pandemic). Hence, the differences were identified in the anxiety from death in Sweden, Italy and the UK before and after the pandemic, as were the differences in their population’s psychological response to the public lockdown policies.

Put differently, our study helps to elucidate (i) the moral role of public institutions as alleviators of mental health negative spillovers and (ii) the cultural differences of them succeeding to do so across space. We illustrate with data that in all countries public institutions do affect the mental health of people through their public health policy. However, the different level of success of the institutions in alleviating the mental health negative externalities in different countries, is due not only to the type of the adopted public policy itself, but also due to the cultural difference in the populations – in this case these are the cultural differences in the propensity of the local populations to experience anxiety from death. We confirm that institutions seem to serve to alleviate public mental pain with their functioning, but how much mental pain they manage to alleviate varies according to the local propensity of the population to feel mental pain regarding the particular problem addressed by the institutions.

Using data from Google trend frequency of search data corresponding to the word” death’, which signify the nation’s anxiety experienced from death, we employ difference-in-differences and interrupted time series analysis approaches to establish the presence of any effects from the public lockdown policy in the UK and Italy. We took advantage of the presence of a natural quasi-experimental setting by including Sweden as a control, because Sweden did not have a public lockdown policy. Our results confirm that the early lockdown in Italy increased anxiety in both Italy and the UK, potentially because asymmetries in policy setting signalled high levels of uncertainty in tackling the Covid-19 pandemic. Consistent with this line of thought, we find that the introduction of the lockdown in the UK reduced anxiety in both the UK and in Italy, perhaps because it confirmed that the consensus is that lockdowns were the right survival strategy.

However, although the present results show a country’s lockdown policies affected its population’s mental health, the effect on mental health of cognitive understanding of daily death toll statistics was more powerful. As these death numbers were higher in Italy, they completely dominated other effects in this country, whereas the public policy in the UK continued to play a role throughout the pandemic. These results were crosschecked both through use of alternative words with connotations relating to anxiety for death (the word” suicide’) and through the use of an emotionally neutral word (the ‘IKEA-effect’ driven word ‘chair’). These robustness checks demonstrate that the narrative economics of language method, suggested by Cultural-Based Development approach, is strong enough to generate evidence for economically meaningful policy, and we showed how this approach can be used to evaluate the impacts of public policy and public statistics on the mental health of the general public. Finally, we presented a detailed analysis of anxiety for death during a representative 24-h window, which showed that the most vulnerable times of the day, when feelings about morbidity are highest, are late in the evening and into the early hours of the morning.

## Appendix: Descriptive statistics

**Table:**

Variable	Obs	Mean	Std. Dev	Min	Max
*Sweden_deaths*	94	10.1	17.8	0	100
*UK_deaths*	94	44.8	18.0	27	100
*Italy_deaths*	94	56.7	13.0	41	100
*deaths_mean_se*	94	3.8	12.2	0	69
*deaths_mean_uk*	94	38.4	119.7	0	685
*deaths_mean_it*	94	156.2	280.9	0	969
**treatment_uk = 0**					
*DIFF_UK_SE*	81	28.5	19.9	-65	68
*DIFF_IT_SE*	81	43.2	20.6	-49	97
*DIFF_IT_UK*	81	14.7	12.2	-14	57
*DIFF_DEATH_UK_SE*	81	2.6	10.6	-1	65
*DIFF_DEATH_IT_SE*	81	59.3	147.1	0	789
*DIFF_DEATH_IT_UK*	81	56.7	139.0	0	737
**treatment_uk = 1**					
*DIFF_UK_SE*	13	73.4	12.1	48	94
*DIFF_IT_SE*	13	68.2	12.9	38	85
*DIFF_IT_UK*	13	-5.2	11.7	-21	20
*DIFF_DEATH_UK_SE*	13	233.8	202.7	17	635
*DIFF_DEATH_IT_SE*	13	732.2	99.7	598	941
*DIFF_DEATH_IT_UK*	13	498.5	217.0	81	788
**treatment_it = 0**					
*DIFF_UK_SE*	70	25.4	18.6	-65	47
*DIFF_IT_SE*	70	42.6	21.1	-49	97
*DIFF_IT_UK*	70	17.3	10.5	2	57
*DIFF_DEATH_UK_SE*	70	0.1	0.3	0	2
*DIFF_DEATH_IT_SE*	70	9.0	28.8	0	168
*DIFF_DEATH_IT_UK*	70	8.9	28.4	0	166
**treatment_it = 1**					
*DIFF_UK_SE*	24	62.1	18.7	18	94
*DIFF_IT_SE*	24	58.4	18.5	19	85
*DIFF_IT_UK*	24	-3.7	10.7	-21	23
*DIFF_DEATH_UK_SE*	24	135.3	183.4	-1	635
*DIFF_DEATH_IT_SE*	24	570.5	231.2	174	941
*DIFF_DEATH_IT_UK*	24	435.3	207.7	81	788

Derived differences between Sweden and Italy and the UK are presented for pre-treatment (pre-lockdown, treatment equal to 0) and during lockdown period (treatment equal to 1)

## Footnotes

1 The suggested parallel with ciometrics here is motivated as follows. Cliometrics can be described as the method of using statistical frequencies for the study of history. Our proposed cliometrics of language here is proposed here as a term to stand for the use of linguistic frequencies for the study of the evolution of socio-economic ‘history’ i.e. socio-economic development broadly defined.

2 This statement clearly links to the literature on mass madness (Reich 1980; Tubadji and Nijkamp 2019; Ulman and Abse 1983), which is also developed as further point in relation to the radicalization of the European states and the narrative economics of language in Tubadji et al. (2020a). Another aspect of mass madness was the toilet paper mania during COVID-19, which resembles the behavioural dynamics of tulip mania (see Garber 1989).

3 Word frequencies have a very heavy-tailed distribution, and can therefore be modeled reasonably well using a Zipf distribution.

4 For more details on this motivation, see Tubadji (2020a,b).

5 There are also other notable approaches to capturing the intangible social aspects of the spread of the pandemic, employing novel methods such as topological data analysis (TDA) (see Dlotko and Rudkin 2020a,b). For the implementation of cultural and institutional analysis on socio-economic development throughout the EU regions see Tubadji and Rudkin (2022).

6 We also undertook a third step where we checked for double-interrupted time series effects due to national and international lockdowns using the ITSA approach (Hahn et al., 2001; Hausman and Rapson, 2018; Heckman et al., 1999; Lee and Lemieux, 2010; McDowall et al., 2019). These results are consistent with the previous results, and are available upon request.

7 An educated guess here might suggest that the low pre-pandemic anxiety from death in Sweden is the reason why the local institutions did not identify a need to make interventions (such as lockdowns) that will serve the reduction of such an anxiety. This interpretation would be in line with the cultural roots of institutions and will line consistently with our further cultural hysteresis findings. This link is an important point of departure for further CBD research on public policy, cultural relativity and cultural hysteresis.

8 We reestimated our ITSA with a double treatment effect, and the results are consistent with those presented Table 2. See footnote 4.

9 This corroborate a tendency for increases in the search intensity for ultra-right preferences across Europe, as revealed by Tubadji et al. (2020a).

10 It might also signal the increase in use of a chair at home and therefore the need to purchase/renew one.
